# Editorial: Mood and Cognition in Old Age

**DOI:** 10.3389/fnagi.2018.00286

**Published:** 2018-09-25

**Authors:** Lia Fernandes, Huali Wang

**Affiliations:** ^1^Department of Clinical Neuroscience and Mental Health, Faculty of Medicine, University of Porto, Porto, Portugal; ^2^Research Unit CINTESIS—University of Porto, Porto, Portugal; ^3^Beijing Dementia Key Lab, National Clinical Research Center for Mental Disorders, Peking University Institute of Mental Health (Sixth Hospital), Beijing, China

**Keywords:** mood disorder, mild cognitive impairment, Alzheimer's disease, neuroimaging, psychology

Depression and cognitive impairment are great challenges for the elderly population. The Research Topic “Mood and Cognition” by Frontiers in Aging Neurosciences comprises 16 articles addressing new findings and perspectives concerning mood and cognition in old age, including age-related disorders (e.g., elderly depression, mild cognitive impairment, and Alzheimer's disease). The articles presented focus on the role of the brain structure and activity in mood and cognition and explore several hypotheses regarding their association with new pathophysiological processes such as inflammation, the association with other comorbidities, as well as the impact of invasive medical procedures on cognition and delirium. Other approaches are focused on the influence of socialization, interpersonal relations, and social learning on cognitive performance and quality of life.

Three articles are focused on the connection between dementia (Alzheimer's Disease-AD) and depression. Liu et al., conducted a study explaining how the hypothalamus interacts with other brain regions in AD patients with depression (D-AD), using a functional connectivity (FC) analysis. This promising study showed that D-AD patients had reduced FC in the hypothalamus, the right middle temporal gyrus and the right superior temporal gyrus compared with the FC of nD-AD patients, and suggested that the abnormal FC between the hypothalamus and the temporal lobe may play a key role in the pathophysiology of depression in AD patients. Lebedeva et al., assessed whether structural brain magnetic resonance imaging (MRI) in late-life depressed patients (LLD) could predict mild cognitive impairment or dementia 1 year prior to the diagnosis. The authors concluded that the analysis of the baseline structural MRI alone was able to accurately distinguish LLD patients developing MCI and dementia, from those remaining cognitively stable. Moreover, the authors showed that the ventral diencephalon, including the hypothalamus, might play an important role in the preservation of cognitive functions in LLD. Following the same line, Liu et al. investigated the relationship among a history of depression, depressive states, and dementia in a community-based old-old cohort in Japan. This valuable study concluded that a history of depression should be considered a risk factor for all-cause dementia and that, in the old-old population, depression was associated with a higher prevalence of dementia, lower cognitive performance, and a smaller hippocampus. The study of Hou et al. examined the implicit relationship between the disruption of interhemispheric functional coordination and cognitive impairment in late-onset depression (LOD), using functional magnetic resonance imaging (fMRI). The authors state that the altered linkage patterns of intrinsic homotopic connectivity and impaired cognitive flexibility may constitute a novel clue regarding the neural substrates underlying cognitive impairment in LOD.

Zhao et al. present a hypothesis and theory article focusing on post-stroke depression (PSD), which is a common neuropsychiatric complication in patients who have suffered a stroke. The authors explored the inflammation hypothesis for PSD and point to preventive and therapeutic strategies, specifically remote ischemic conditioning (RIC). They concluded that RIC may be a novel approach to prevent PSD, with potential to be widely used in clinical practice and to be applied in other neurobehavioral disorders.

Moretti and Signori conducted a very interesting review regarding the neural correlates for apathy in frontal-prefrontal and parietal cortical-subcortical circuits. In spite of the controversial definitions, with different categorizations of this nosographical entity, the present discussion may contribute to new insights about apathy in the context of several pathologies being degenerative, vascular, acute, or chronic. The authors concluded by highlighting important future directions toward goal-specific problems.

In their article Gamaldo et al. examine the rates, predictors, and outcomes of sleep disturbances in older hospitalized patients. This is the first study to use a large national (USA) healthcare database, with 35,258,031 of older adults. Stating that the proportion of older adults with a sleep diagnosis has increased significantly over the last decade, this study documented an important association between increasing sleep disturbance rates and expenditures within hospital settings. Moreover, co-morbidities such as depression, cardiovascular risk factors, and neurological disorders steadily increased over time in these patients. Also from the USA, Assari and Lankarani conducted a study focused on the reciprocal and longitudinal associations between depressive symptoms and mastery, comparing black and white American older adults. They found that, among white but not black older adults, higher levels of depressive symptoms at baseline predicted a greater decline in sense of mastery over 3 years' follow-up, stating that race may alter how depression is linked to changes in evaluation of self (e.g., mastery) over time.

Focusing on cognition, the article of Palaci et al. explored how parental economic socialization affects the planning for retirement (FPR) through the mediation of financial literacy, financial planning decisions, and financial management. The results show that parental economic socialization directly and indirectly influences FPR, and that parental economic behavior acts as a positive model for the development of financial literacy and skills and for decisions about FPR. Based on this, the authors point out important future lines of research.

Ouanes et al. conducted a population-based study, hypothesizing that increased cortisol may be associated with poorer cognition and with certain personality traits (mainly high neuroticism), and that personality might explain the association between cortisol and cognition. This study found that high agreeableness and openness might be associated with poorer executive performance in later life. Moreover, increased cortisol may be associated with both specific personality traits (high extraversion, low openness) and worse cognitive performance. In spite of this, the authors concluded that the association between personality traits and cognitive impairment seems to be independent of increased cortisol production and its effects on cognition.

Two articles are specifically dedicated to Alzheimer Disease (AD). The article from Wang et al. explored the interaction dynamics between different electroencephalographic (EEG) oscillations in AD patients, comparing the resting eye-closed EEG signals in AD patients and healthy volunteers. The authors propose that the pathological increase of ongoing gamma-band power might result from the disruption of the GABAergic interneuron network in AD patients. They also suggest that the cross-frequency overcouplings, might indicate the attenuated complexity of the neuronal network, and that AD patients have to use more neural resources to maintain the resting brain state. These promising findings provide new evidence of the disturbance of the brain oscillation network in AD and further deepen the understanding of the central mechanisms of AD. Focused on the genetics of the disease, Lukiw et al. provide recent evidence for the mis-regulation of a small family of genes expressed in the human hippocampus that appear to be significantly involved in the expression patterns common to both AD and aggression. The magnitude of genes expression implicated in aggressive behavior appears to be more pronounced in the later stages of AD when compared to MCI. These recent genetic data further indicate that the extent of cognitive impairment may have some bearing on the degree of aggression which accompanies the AD phenotype.

Two other important articles focused on delirium. El-Gabalawy et al. developed a novel stress-diathesis model based on comprehensive pre-operative psychiatric and neuropsychological testing, a blood oxygenation level-dependent (BOLD) magnetic resonance imaging (MRI) carbon dioxide (CO_2_) stress test, and high fidelity measures of intra-operative parameters that may interact facilitating post-operative delirium (POD). Results provide preliminary support for the interacting of diatheses (vulnerabilities) and intra-operative stressors on the POD phenotype. Based on these initial findings, the authors offer some recommendations for intra-operative management for patients at risk of POD. The article from Dong et al. presents the results of a clinical investigation on the associations between the preoperative expression levels of microRNA (miR)-146a, miR-125b, and miR-181c in cerebrospinal fluid (CSF) and serum and the development and severity of post-operative delirium (POD). It found that dysregulation of preoperative miR-146a and miR-181c in CSF and serum was associated with the development and severity of POD, and that these NeurimmiRs might participate in the neuropathogenesis of POD.

Also exploring cognitive changes after invasive medical procedures, Kulason et al. present preliminary results of a pilot study, being the first report to examine Postoperative Cognitive Decline (POCD) after major thoracic surgery (partial pulmonary lobectomy lung) in elderly Japanese patients. This pioneer study clarified that decline in cognition is detectable to a certain extent after the surgery. Additionally, longer anesthetic exposure may negatively impact on attention and working memory, and preoperative mental well-being is a possible predictor of POCD.

Cleutjens et al. investigated whether macrostructural brain MRI features of cerebral small vessel disease (SVD) and hippocampal volume (HCV) are related to cognitive performance in patients with chronic obstructive pulmonary disease (COPD). No group differences were reported, regarding demographics, clinical characteristics, comorbidities and the presence of SVD features and HCV. This way, the authors concluded that there is not yet evidence for a relationship between cerebral SVD and HCV and cognitive functioning in patients with COPD and that additional studies will be needed to determine other possible mechanisms of cognitive impairment in these patients.

In all, this Research Topic may contribute to building different perspectives on how mood and cognition in late life could be influenced by the brain structure, activity and aging, and how this may interact with environmental stimuli and interpersonal relationships. All these studies would enrich our understanding of the bio-psycho-social mechanisms underlying improving psychological well-being and cognitive health.

## Author contributions

All authors listed have made a substantial, direct and intellectual contribution to the work, and approved it for publication.

### Conflict of interest statement

The authors declare that the research was conducted in the absence of any commercial or financial relationships that could be construed as a potential conflict of interest.

